# What’s in a name? A comparison of attitudes towards artificial intelligence (AI) versus augmented human intelligence (AHI)

**DOI:** 10.1186/s12911-020-01158-2

**Published:** 2020-07-20

**Authors:** Santiago Romero-Brufau, Kirk D. Wyatt, Patricia Boyum, Mindy Mickelson, Matthew Moore, Cheristi Cognetta-Rieke

**Affiliations:** 1 Mayo Clinic Kern Center for the Science of Healthcare Delivery, Mayo Clinic, 200 First Street SW, Rochester, MN 55905 USA; 2 Department of Biostatistics, Harvard T. H. Chan School of Public Health, Harvard University, 655 Huntington Avenue, Boston, MA 02115 USA; 3 Division of Pediatric Hematology/Oncology, Department of Pediatric and Adolescent Medicine, Mayo Clinic, Rochester, MN USA; 4 Department of Nursing, Mayo Clinic Health System, La Crosse, WI USA

**Keywords:** Intelligence, artificial, Terminology, Attitude of health personnel, Clinical decision making, Decision making

## Abstract

**Background:**

“Artificial intelligence” (AI) is often referred to as “augmented human intelligence” (AHI). The latter term implies that computers support—rather than replace—human decision-making. It is unclear whether the terminology used affects attitudes and perceptions in practice.

**Methods:**

In the context of a quality improvement project implementing AI/AHI-based decision support in a regional health system, we surveyed staff’s attitudes about AI/AHI, randomizing question prompts to refer to either AI or AHI.

**Results:**

Ninety-three staff completed surveys. With a power of 0.95 to detect a difference larger than 0.8 points on a 5-point scale, we did not detect a significant difference in responses to six questions regarding attitudes when respondents were alternatively asked about AI versus AHI (mean difference range: 0.04–0.22 points; *p* > 0.05).

**Conclusion:**

Although findings may be setting-specific, we observed that use of the terms “AI” and “AHI” in a survey on attitudes of clinical staff elicited similar responses.

## Background

In 2018, the American Medical Association released a policy statement on augmented intelligence in medicine [1]. The wording “augmented intelligence” was carefully chosen in contradistinction to the more colloquial term “artificial intelligence” (AI) to emphasize that while computing systems have the capability to augment human medical decision making, these systems are not a replacement for rational human thought.

Popular culture and science fiction are plagued with examples of AI as a competing intelligence to be feared, and a recent survey of attitudes about AI among the general public found that only a minority support the development of AI [2]. In fact, most Americans believed that automation and AI would result in a net destruction of jobs [2]. Perhaps in response to these public perceptions, major cloud computing vendors, including IBM [3] and Microsoft [4] also have stated a preference to refer to the technology as “augmented” rather than “artificial” intelligence. Although the idea of “augmented human intelligence” (AHI) has been around for over 50 years, [5] the term “artificial intelligence” continues to be the prevailing term that is used. At our own institution, Mayo Clinic has established “augmented human intelligence” (AHI) as the preferred term, [6] yet our experience has been that in practice the two terms are often used interchangeably.

Perceptions of technology and attitudes toward these technologies are key elements that can affect the uptake and success of implementation. However, there is a lack of evidence as to whether there is a measurable difference in perceptions and attitudes toward the technology when it is referred to as “augmented human intelligence” versus “artificial intelligence” among health care staff. Therefore, in this study we sought to understand whether use of the less frequently used—but institutionally-preferred—term “augmented human intelligence” led to more favorable staff perceptions and attitudes about the technology.

## Methods

### Ethics review

This study was reviewed by the Mayo Clinic Institutional Review Board and deemed “exempt.” No patient identifiable information was used.

### Survey participants and clinical context

The study was performed in three regional health system primary care clinics and a 250-bed hospital in a medium-sized city in the American Midwest where AI/AHI was not routinely used as part of clinical care. An electronic survey was emailed in the context of a quality improvement project that included implementation of two decision support systems powered by AI/AHI. One system aimed to identify outpatients with diabetes mellitus who were at risk for poor glycemic control and intervene to reduce that risk (“diabetes-related AI/AHI”). The other system aimed to identify inpatients who were at high risk for hospital readmission and to intervene to reduce that risk (“hospital readmission-related AI/AHI”). Survey participants were clinical staff (physicians, nursing staff and other allied health staff) in the clinics that had been selected to participate in the pilot project. There was a pre-implementation survey (not reported herein) in which the technology was referred to using the common term, “artificial intelligence”. During the initial presentations of the project to frontline clinical staff, neither of the two terms (i.e., AI or AHI) were used; instead, the technology was primarily referred to as “predictive analytics” or “cognitive computing.” The following subsection describes the survey of attitudes.

### Survey questions

The survey included six questions, which respondents were asked to respond to using a 5-point Likert scale:

- I am generally familiar with (AI/AHI).
- I routinely use (AI/AHI) support in my job.
- I am excited about how (AI/AHI) can help me with my job.
- I am worried that (AI/AHI) will make my job more complicated.
- I am worried that (AI/AHI) will make my job obsolete.
- I believe (AI/AHI) will not be able to understand my job well enough to help.

Survey participants were randomized to have questions worded with the term “artificial intelligence” or “augmented human intelligence.” Participants randomized into each group were emailed an invitation to complete the survey. In addition to the above questions, participants were asked to self-report their role (e.g., physician, registered nurse, other allied health staff role).

### Statistical analysis

Statistical analysis was performed using JMP (Sas Institute, Cary, North Carolina) and Microsoft Excel (Microsoft, Seattle, Washington). Student’s t-test was used to assess for a difference in Likert scale responses [7]. P-values < 0.05 were considered statistically significant. Power analysis revealed that combined analysis of the diabetes-related and hospital admission-related AHI questions had a power of 0.95 to detect a difference larger than 0.8 points on a 5-point scale, and a power of 0.8 to detect a difference of 0.65 on a 5-point scale.

## Results

Thirty-seven participants of the diabetes-related AI/AHI pilot and 56 of the hospital readmission-related AI/AHI pilot completed the survey (Table 1). The response rate was 46% for staff involved with diabetes-related AI/AHI and 38% for staff involved with hospital readmission-related AI/AHI, yielding an overall response rate of 41% (Table 1).

**Table 1:** Survey respondent demographics

Attribute	Diabetes-related		Hospital readmission-related	
	AI	AHI	AI	AHI
**Surveys sent**	41	40	73	73
**Surveys completed**	18	19	29	27
**Work Role**				
Physician	4	7	3	6
Nurse	6	9	17	11
Other allied health staff	8	3	9	10

**Fig. 1:**
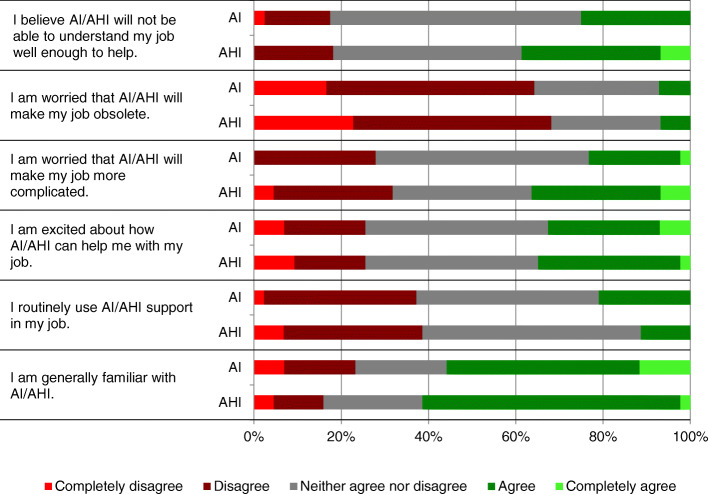
Survey responses

Survey responses are shown numerically in Table 2 and graphically in Fig. 1. Mean response score differences ranged from 0.04 to 0.22 points out of 5. No statistically significant difference was observed when comparing responses between respondents who were asked about AI versus AHI within each group (i.e., diabetes-related vs. hospital readmission related) or when combining the two groups (i.e., *p* > 0.05). At least 60% of respondents in each group did not think that AI or AHI would make their jobs obsolete. Respondents were largely ambivalent about the ability of AHI to understand their jobs.

**Table 2:** Summary of survey responses

	% (n)	Completely disagree	Disagree	Neither agree nor disagree	Agree	Completely agree
I believe AI/AHI will not be able to understand my job well enough to help.	AI (*n* = 40)	3% (1)	15% (6)	58% (23)	25% (10)	0% (0)
	AHI (*n* = 44)	0% (0)	18% (8)	43% (19)	32% (14)	7% (3)
I am worried that AI/AHI will make my job obsolete.	AI (*n* = 42)	17% (7)	48% (20)	29% (12)	7% (3)	0% (0)
	AHI (n = 44)	23% (10)	45% (20)	25% (11)	7% (3)	0% (0)
I am worried that AI/AHI will make my job more complicated.	AI (*n* = 43)	0% (0)	28% (12)	49% (21)	21% (9)	2% (1)
	AHI (*n* = 44)	5% (2)	27% (12)	32% (14)	30% (13)	7% (3)
I am excited about how AI/AHI can help me with my job.	AI (*n* = 43)	7% (3)	19% (8)	42% (18)	26% (11)	7% (3)
	AHI (*n* = 43)	9% (4)	16% (7)	40% (17)	33% (14)	2% (1)
I routinely use AI/AHI support in my job.	AI (n = 43)	2% (1)	35% (15)	42% (18)	21% (9)	0% (0)
	AHI (*n* = 44)	7% (3)	32% (14)	50% (22)	11% (5)	0% (0)
I am generally familiar with AI/AHI	AI (*n* = 43)	7% (3)	16% (7)	21% (9)	44% (19)	12% (5)
	AHI (*n* = 44)	5% (2)	11% (5)	23% (10)	59% (26)	2% (1)

## Discussion

At our institution, survey responses about attitudes following use of augmented human intelligence did not appear to vary when respondents were asked about AI or AHI. We were reassured by this, as we were worried that staff may misunderstand questions if asked using the institutionally-preferred term “AHI” rather than the more common term “AI.”

The finding that survey responses did not vary based on wording also suggests that staff perceived these two terms similarly. Although we did not directly assess perceptions of the two terms (e.g., by asking “How are AI and AHI different?”), this finding suggests that use of the term AHI does not necessarily “soften” attitudes or yield more favorable attitudes. This is similar to the findings of a survey of attitudes toward AI conducted in the general population [2], which compared attitudes toward “AI” and “machine learning” and found similar results for both terms.

One of the possible limitations of any negative study is whether it was powered to detect a difference that would be practically significant. Based on our sample size, we had a power of 95% to detect a difference greater than 0.8 points in our 5-point Likert scale, and an 80% power to detect a difference greater than 0.65 points. Although there is a possibility that a smaller difference existed but was not detected, we deemed differences less than one point to likely be of little practical significance.

However, we may have failed to detect variation in different attitudinal dimensions, and our study was not sufficiently powered to conduct sub-group analyses to determine whether attitudes differed between clinical work roles. We also only assessed the attitudes of staff at a regional health system clinic where a specific AI/AHI-based pilot project was being implemented, leaving the possibility that attitudes may vary in other settings where AI/AHI was not being implemented. In an initial survey, the common term “AI” was used. We cannot rule out the possibility that use of this term led to anchoring and contributed to the lack of an observed difference in responses between the groups. However, our observation that approximately 40% of respondents in reach group did not report being familiar with AI/AHI suggests that this may not have been the case for some staff. Indeed, it is possible that lack of familiarity and deep understanding of the terms (AI/AHI) accounts for the observation that there was no difference in responses when the two terms were used. During the project, we avoided using the term “AHI.” When this term was introduced in the survey presented in this manuscript, it did not seem to soften staff attitudes toward the technology, as some have hypothesized it would [3, 4, 6]. Attitudes of clinical staff elsewhere and of the general population (i.e., patients) may differ. Finally, because we targeted a convenience sample of staff participating in a quality improvement project, we must consider the degree to which selection bias may have influenced participant responses.

Our finding that attitudes did not appear to be different when technology was referred to as “AI” versus “AHI” can yield different interpretations. One interpretation is that the terms are perceived equivalently and can both be used interchangeably. An alternative interpretation is that, because the choice of term does not meaningfully influence staff’s perceptions about the work domain, other considerations should dictate which term is preferred. Our institutional leadership has chosen “augmented human intelligence” (“AHI”) as the institutionally-preferred term, rather than “artificial intelligence” (“AI”). To maintain consistency, we will utilize the institutionally-preferred in future pilot projects with clinical staff.

## Conclusions

Although findings may be setting-specific, we observed that use of the terms “AI” and “AHI” in a staff survey on attitudes towards machine learning-based clinical decision support elicited similar responses.

## Data Availability

The dataset is not publicly available because the Institutional Review Board application did not include provisions for sharing data with external parties.

## Abbreviations

AI: Artificial Intelligence
AHI: Augmented Human Intelligence
